# Leveraging machine learning models to forecast pediatric allergic rhinitis exacerbation risk based on environmental exposure data

**DOI:** 10.3389/fped.2026.1865399

**Published:** 2026-07-22

**Authors:** Zhicheng Li, Shan Huang, Zhongfang Xia

**Affiliations:** Department of Otolaryngology, Wuhan Children’s Hospital, Tongji Medical College, Huazhong University of Science and Technology, Wuhan, Hubei, China

**Keywords:** allergic rhinitis, machine learning, pediatric, PM2.5, prediction model, *Shap*, TRIPOD+AI

## Abstract

**Objective:**

Acute exacerbations of pediatric allergic rhinitis (AR) are difficult to anticipate, and individualized risk tools integrating clinical and environmental data are lacking. We developed an interpretable machine-learning model to predict 90-day AR exacerbation risk in children and conducted a preliminary transportability assessment in an independent cohort, following TRIPOD + AI guidelines.

**Methods:**

A development cohort (*n* = 2,000) was assembled by linking NHANES (2005–2006 and 2011–2018 cycles) with EPA Air Quality System and NOAA meteorological data; an independent external cohort (*n* = 50) was recruited at Wuhan Children's Hospital (2023–2024). Clinical predictors [Total Nasal Symptom Score (TNSS), total IgE, eosinophils, comorbidities] and environmental exposures (same-day and 0–7-day-lag PM_2.5_, PM_10_, O_3_, NO_2_, temperature, humidity) were analyzed. L2-regularized logistic regression (LR) was prespecified as the primary model and benchmarked against random forest, XGBoost, LightGBM, and SVM using nested 5-fold cross-validation and multiple imputation (m = 5). Discrimination, calibration, decision-curve analysis, and SHAP interpretability were evaluated.

**Results:**

Exacerbation occurred in 34.4% (687/2,000) of the development cohort and 36.0% (18/50) of the external cohort. The primary LR model achieved an internal AUC of 0.81 (95% CI: 0.74–0.88) and an external AUC of 0.71 (95% CI: 0.50–0.92); the wide confidence interval reflects the limited precision of the small validation sample. Baseline TNSS, same-day PM_2.5_, and total IgE were the most influential predictors across feature-selection and SHAP analyses. Children exposed to PM_2.5_ > 75 μg/m^3^ had approximately five-fold higher exacerbation odds than those exposed to <35 μg/m^3^ (OR = 5.08, 95% CI: 3.45–7.47). A sensitivity model excluding TNSS retained moderate discrimination (external AUC 0.66, 95% CI: 0.49–0.83), supporting the independent contribution of environmental and immunologic factors. Decision-curve analysis showed positive net benefit across threshold probabilities of 0.10–0.35.

**Conclusion:**

An interpretable LR model integrating baseline symptom burden with ambient PM_2.5_ exposure demonstrated promising predictive performance for pediatric AR exacerbation. However, the small external cohort (*n* = 50) yielded wide confidence intervals, and findings should be regarded as preliminary transportability evidence rather than definitive external validity. Larger multicenter prospective studies with site-specific recalibration are required before clinical implementation.

## 1 Introduction

Allergic rhinitis (AR) is one of the most prevalent chronic respiratory diseases in children, affecting approximately 10–40% of the pediatric population globally (1), with increasing prevalence rates observed in developing countries over recent decades (2). The condition is characterized by nasal congestion, rhinorrhea, sneezing, and nasal itching, triggered by IgE–mediated inflammatory responses to environmental allergens such as pollen, dust mites, animal dander, and mold spores (3). The pathophysiology involves sensitization to specific allergens, leading to mast cell activation and release of inflammatory mediators including histamine, leukotrienes, and prostaglandins that cause the characteristic symptoms (4). Acute exacerbations of AR not only significantly impair children's quality of life, affecting sleep quality, school performance, and social interactions (5), but also substantially increase healthcare utilization through emergency department visits, unscheduled clinic appointments, and medication escalation (6). Furthermore, AR is frequently associated with comorbidities such as asthma (the “unified airway” concept) (7), allergic conjunctivitis (8), atopic dermatitis, and chronic rhinosinusitis, creating a complex disease burden that requires comprehensive management strategies.

Environmental factors play a crucial role in the pathogenesis and exacerbation of AR, with mounting epidemiological and mechanistic evidence supporting the adverse effects of ambient air pollution on allergic airway diseases (9). Fine particulate matter (PM_2.5_), defined as particles with aerodynamic diameter less than 2.5 micrometers, has been increasingly recognized as a significant trigger for AR symptoms and exacerbations due to its ability to penetrate deep into the respiratory tract and even enter systemic circulation (10). Other pollutants including nitrogen dioxide (NO_2_), ozone (O_3_), sulfur dioxide (SO_2_), and coarse particulate matter (PM_10_) have also been implicated in allergic airway inflammation (11). The mechanisms through which these pollutants exacerbate AR are multifaceted: they can enhance allergic sensitization by acting as adjuvants to allergens (12), amplify Th2–mediated inflammatory responses through oxidative stress pathways (13), directly damage the nasal epithelial barrier function leading to increased allergen penetration (14), and promote the release of pro–inflammatory cytokines including IL–4, IL–5, IL–13, and IL–33 (15). Additionally, meteorological factors including temperature extremes, rapid temperature changes, and humidity variations may independently influence AR symptom severity through effects on allergen distribution (pollen counts, mold spore concentrations), mucociliary clearance efficiency, and epithelial barrier integrity (16). The complex interplay between multiple environmental exposures and individual susceptibility factors creates significant challenges for clinical prediction and management.

Despite the established epidemiological associations between environmental exposures and AR exacerbations, clinicians currently lack reliable tools to predict individual exacerbation risk in their daily practice (17). This gap between population–level evidence and personalized prediction represents a critical unmet clinical need. Traditional statistical approaches, including conventional logistic regression and Cox proportional hazards models, often fail to adequately capture the complex, non–linear, and potentially interactive relationships between multiple risk factors and clinical outcomes (18). These methods typically require pre–specification of variable transformations and interactions, which may not optimally represent the true underlying data structures. Machine learning (ML) algorithms offer promising solutions to this challenge by automatically learning complex patterns from high–dimensional data without requiring explicit feature engineering (19). These algorithms can leverage sophisticated pattern recognition capabilities to integrate diverse data types–including clinical measurements, laboratory values, environmental exposures, and patient demographics–and identify intricate predictor–outcome relationships that may be missed by traditional approaches (20). The flexibility of ML methods in handling non–linear effects, high–order interactions, and heterogeneous data makes them particularly suitable for medical prediction tasks where the underlying biology is complex and incompletely understood (21).

Recent advances in ML applications in respiratory medicine have demonstrated the potential of these approaches for disease prediction and risk stratification (22). However, studies specifically focusing on pediatric AR exacerbation prediction using combined clinical and environmental data remain limited. Furthermore, the interpretability of ML models–critical for clinical translation and trust–has often been overlooked in previous research (23).

**Figure 1:**
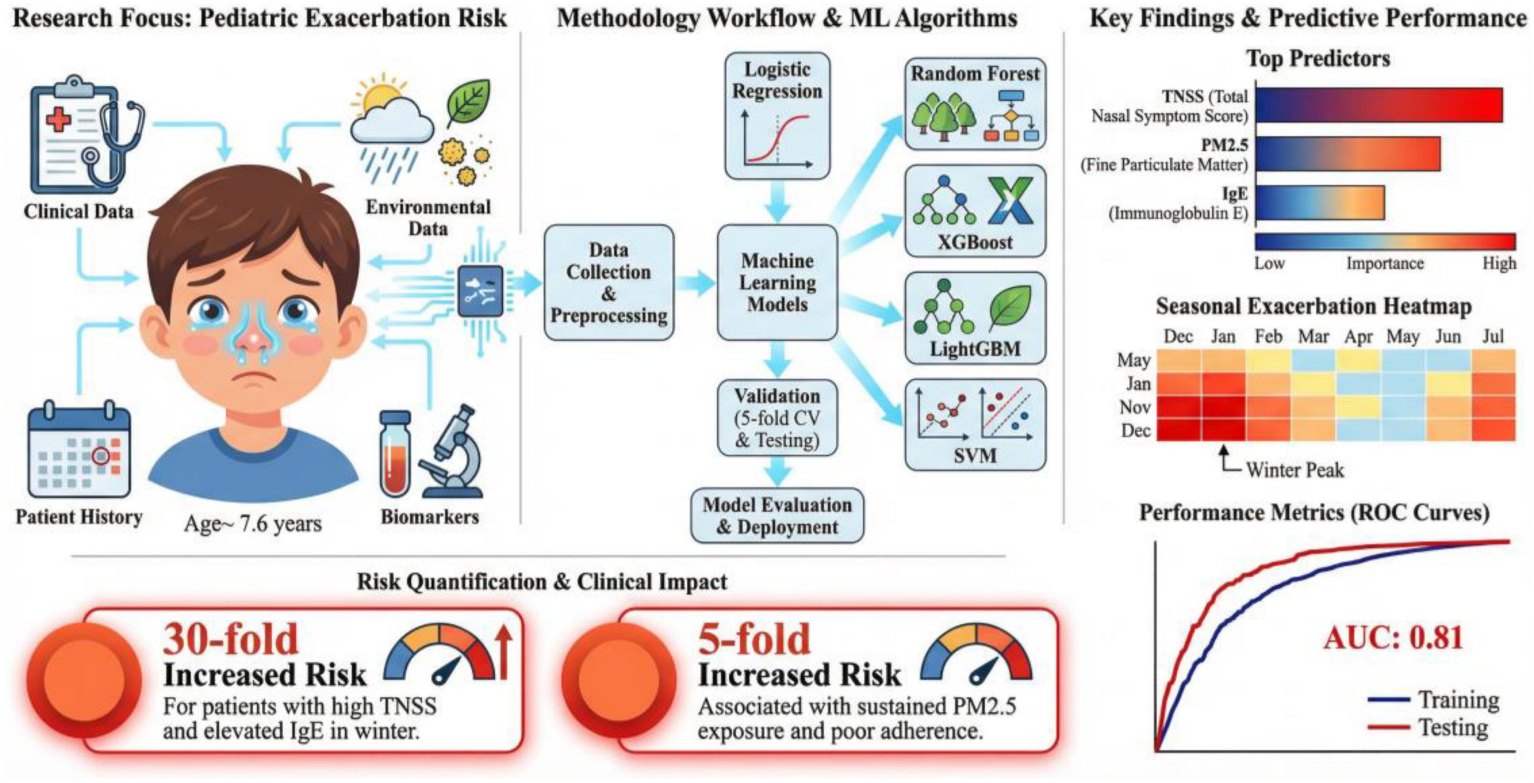
Schematic diagram of the study framework.

In this study, we aimed to develop and externally validate interpretable machine learning models for predicting acute exacerbation risk in pediatric AR patients by integrating baseline clinical features with ambient environmental exposure data, with prespecified sensitivity analyses to evaluate the marginal predictive contribution of symptom burden versus environmental exposures. The overall study workflow–including data source linkage, cohort construction, modeling pipeline, internal and preliminary transportability assessment, and interpretability analyses–is illustrated in Figure 1. All analyses followed the TRIPOD + AI (Transparent Reporting of a multivariable prediction model for Individual Prognosis Or Diagnosis, extension for artificial intelligence) reporting guideline (24); the completed checklist is provided in Supplementary Material S1. Logistic regression was prespecified as the primary model on the basis of parsimony and favorable external behavior in prior respiratory-disease prediction studies, with tree-based and kernel methods serving as pre-registered comparators. SHapley Additive exPlanations (SHAP) were used to interpret the primary model and to explore effect modification between clinical and environmental predictors.

## 2 Methods

### 2.1 Study design and data sources

This retrospective cohort study, reported in accordance with TRIPOD + AI (24) and the RECORD extension for routinely collected data (25), integrated population-level publicly available data for model development with an independent hospital-based cohort for preliminary transportability assessment. The development cohort (*n* = 2,000) was assembled through deterministic record linkage of three data sources: (i) the National Health and Nutrition Examination Survey (NHANES, 2005–2006 and 2011–2018 cycles) (26), which provided demographic characteristics, allergy-related questionnaires (Medical Conditions Questionnaire items MCQ010, MCQ035 and MCQ053), serum total and specific IgE (where available in laboratory files), complete blood count with differential (peripheral eosinophils), household smoking exposure (SMQFAM), pet exposure items, and asthma/atopic dermatitis/allergic conjunctivitis history; (ii) the United States Environmental Protection Agency (EPA) Air Quality System, which supplied daily gravimetric PM_2.5_, PM_10_, and mean ozone and NO_2_ concentrations from ground-based regulatory monitors; and (iii) the National Oceanic and Atmospheric Administration (NOAA) Global Summary of the Day (GSOD) dataset, from which daily mean temperature and relative humidity were obtained. Because participant residential address is a restricted-access variable in NHANES, spatiotemporal linkage was performed under an approved NCHS Research Data Center (RDC) protocol: for each eligible child, the nearest monitor within the same county was identified, and the daily exposure on the self-reported examination date was assigned as the “same-day” exposure, with lagged exposures (lag 1–7 days) computed analogously. A variable-to-source mapping and the NHANES extraction logic are provided in Supplementary Table S1. Where a required laboratory measurement (e.g., quantitative total IgE) was not available in a given NHANES cycle, the variable was treated as missing and imputed as described in Section 2.6.

Eligible NHANES participants were children aged 3–14 years who answered affirmatively to the questionnaire items corresponding to physician-diagnosed allergic rhinitis/hay fever and who had at least one positive specific IgE result in the available allergy panel, consistent with prior NHANES-based definitions of allergic sensitization (26). Of 4,128 potentially eligible NHANES participants, 2,128 were excluded for incomplete residential-county information, failed monitor linkage, or missing primary clinical covariates (inclusion flow in Figure S1), yielding the analytic development cohort of *n* = 2,000.

The external transportability cohort (*n* = 50) consisted of consecutively enrolled pediatric AR patients aged 3–14 years at Wuhan Children's Hospital (WCH), Wuhan, Central China, between March 2023 and November 2024. Diagnosis of AR was established by board-certified otolaryngologists or pediatric allergists using ARIA criteria (27) with either positive skin prick testing or serum-specific IgE against at least one common aeroallergen. Clinical, laboratory and environmental exposure data were collected prospectively using study-specific case-report forms. This cohort provided geographic, temporal and healthcare-system-level preliminary transportability assessment, given substantially different ambient exposure distributions and aeroallergen spectra in Central China compared with the United States. The study was approved by the Institutional Review Board of WCH, and written informed consent was obtained from parents or legal guardians, with assent obtained from children ≥7 years. Use of NHANES and linked federal data was performed in accordance with NCHS Research Ethics Review Board protocols. Because the external cohort was intentionally designed as an initial independent assessment of geographic transportability, its sample size was limited. Therefore, external performance estimates should be interpreted primarily as exploratory evidence of model transportability rather than definitive validation of generalizability.

### 2.2 Study population

Eligible participants were children aged 3–14 years with physician-diagnosed allergic rhinitis, defined by compatible clinical history and evidence of allergic sensitization (skin-prick testing and/or positive specific IgE). Children with concurrent acute respiratory infection within the preceding two weeks, systemic corticosteroid use within the preceding four weeks, or a chronic respiratory disease other than asthma were excluded. Participants were required to have non-missing values for the pre-specified minimum predictor set (age, sex, baseline TNSS, total IgE, asthma status, same-day PM_2.5_, and index date); missingness in secondary predictors was handled by multiple imputation (Section 2.6). For all enrolled subjects, demographic characteristics, disease duration, family and personal history of allergy, and comorbidities were systematically documented at the index encounter.

### 2.3 Clinical assessments

All clinical predictors were defined as baseline measurements obtained at the index encounter, which was set prior to, and independent of, any subsequent exacerbation event (see Section 2.5). The baseline Total Nasal Symptom Score (TNSS) was calculated as the sum of four symptom domains (nasal congestion, rhinorrhea, sneezing, and nasal itching), each rated on a 0–3 scale for a total range of 0–12 (28), reflecting the child's usual symptom burden during the two weeks preceding the index encounter rather than acute-episode symptoms. A Visual Analog Scale (VAS) of overall baseline symptom severity ranging from 0 to 10 was recorded in parallel (29). Baseline laboratory evaluations comprised serum total IgE (IU/mL), the number of positive specific IgE results (aeroallergen panel including house dust mite, cat dander, dog dander, mold mix, and regional pollens), and peripheral blood eosinophil counts (×10⁹/L). Comorbidities (asthma, allergic conjunctivitis, and atopic dermatitis) and lifestyle factors (indoor smoke exposure, pet exposure, and air purifier use) were systematically recorded. To minimize circularity between baseline symptom measurement and the exacerbation outcome, baseline TNSS was recorded at the index encounter prior to outcome ascertainment. In the Wuhan cohort, TNSS measurements were obtained during clinically stable visits, defined as the absence of unscheduled visits or rescue medication use within the preceding 14 days. Because NHANES is a cross-sectional survey without longitudinal event tracking, equivalent verification was not possible in the development cohort, and baseline symptom measurements should be interpreted as survey-based assessments obtained at the examination visit.

### 2.4 Environmental exposure assessment

Environmental exposures were assigned at the residential ZIP-code level using regulatory-monitor data from the EPA AQS (United States) and from the Ministry of Ecology and Environment national air-quality network (China, for WCH). Air-quality parameters included PM_2.5_ (*μ*g/m^3^), PM_10_ (μg/m^3^), ozone (O_3_, μg/m^3^) and nitrogen dioxide (NO_2_, μg/m^3^). Meteorological variables (daily mean temperature,°C; relative humidity, %) were obtained from NOAA GSOD. For each participant the following exposure metrics were precomputed: (i) same-day mean (index date), (ii) lag 1–7 day means, and (iii) 7-, 14- and 30-day cumulative averages. The primary exposure of interest was the same-day PM_2.5_ concentration at the index encounter; lagged and cumulative windows were examined in sensitivity analyses. To address pollen-mediated triggers not directly captured by ambient regulatory monitors, monthly pollen-load indices (tree, grass and weed) were extracted from the National Allergy Bureau (NAB) for U.S. locations and from published regional pollen calendars for central and eastern China applicable to Wuhan (30), and entered as categorical predictors (low/medium/high/very high). Joint effects of correlated pollutants were additionally assessed by weighted quantile sum (WQS) regression (31) as a sensitivity analysis, to complement the single-pollutant machine-learning framework. Because harmonized daily pollen-count data were not available across all study regions and years, monthly pollen-load indices were used as surrogate indicators of seasonal aeroallergen burden. These variables were intended to capture broad seasonal patterns rather than short-term aeroallergen fluctuations.

### 2.5 Outcome definition

The primary outcome was an objectively ascertained acute exacerbation of AR occurring within a 90-day follow-up window beginning on the day after the index encounter. An exacerbation event was defined by the occurrence of at least one of the following, ascertained from administrative and electronic health-record data rather than from a repeat TNSS measurement: (i) an unscheduled outpatient visit with a primary or secondary International Classification of Diseases (ICD-10) code of J30.x for AR; (ii) an emergency-department attendance for AR-related symptoms; or (iii) a documented escalation of AR-directed pharmacotherapy beyond the patient's baseline regimen (e.g., addition of an intranasal corticosteroid, oral antihistamine, or leukotriene receptor antagonist, or a short course of oral corticosteroids). For the purposes of this study, medication escalation was operationally defined as any physician-documented addition of a new AR-directed therapeutic class beyond the baseline treatment regimen, including initiation of an intranasal corticosteroid, oral second-generation antihistamine, leukotriene receptor antagonist, combination therapy involving two or more classes, or a short course of systemic corticosteroids for uncontrolled symptoms. Simple dose adjustments within the same medication class were not considered escalation events. To avoid outcome-by-predictor circularity, the exacerbation definition did not include any post-index TNSS threshold; the baseline TNSS (Section 2.3) was captured at least 14 days before any qualifying event and was therefore temporally antecedent to the outcome. In patients with concomitant asthma, events were adjudicated by two independent reviewers blinded to model predictions as (a) AR-predominant, (b) asthma-predominant, or (c) mixed, based on the dominant symptom complex, medication changes and coding; only AR-predominant and mixed events contributed to the primary outcome, and asthma-predominant events were analyzed in a prespecified sensitivity analysis. Inter-rater agreement was assessed by Cohen's *κ*, with disagreements resolved by a third senior adjudicator. To improve consistency of outcome ascertainment, medication-escalation events were evaluated against ARIA guideline-based treatment principles. In the Wuhan cohort, treatment decisions followed institutional protocols derived from ARIA recommendations. In the NHANES-linked cohort, escalation events were identified using predefined medication-class changes rather than physician-specific prescribing preferences whenever possible.

### 2.6 Model development

Five algorithms were benchmarked: L2-regularized logistic regression (LR), random forest (32), XGBoost (33), LightGBM (34) and support vector machine (SVM) with radial basis function kernel (35). LR was prespecified as the primary model on the basis of parsimony, transparency, and favorable reported external behavior in comparable clinical prediction tasks (20, 36); the remaining four models were treated as pre-registered comparators. Categorical variables were one-hot encoded, continuous variables were standardized to zero mean and unit variance (for LR and SVM) or left on their native scale (for tree-based models), and counts of positive sIgE and exposure metrics were retained on their original numeric scales. Missing predictor values (complete-case rate: 71.8% in the development cohort, 94.0% in WCH) were handled by multiple imputation by chained equations (MICE, m = 5), with predictive mean matching for continuous variables and logistic regression for binary variables (37); models were fitted on each imputed dataset and pooled following Rubin's rules. Class imbalance (34.4% event rate) was addressed by inverse-prevalence class weighting rather than oversampling, to preserve the calibration of predicted probabilities (38).

Feature screening was performed inside the cross-validation loop to avoid optimistic bias: within each training fold, (a) LASSO logistic regression (39) with the regularization parameter *λ* selected by minimum one-standard-error cross-validated deviance, and (b) permutation-based random-forest importance were computed, and a feature was retained if it had a non-zero LASSO coefficient or fell within the top-15 permutation importance. Hyperparameters were tuned using nested 5-fold cross-validation on the development cohort, optimizing the mean cross-validated AUC. The searched grids were: LR [L2 penalty C ∈ (0.01, 0.1, 1, 10)]; random forest [number of trees ∈ {200, 500, 1000}, max depth ∈ {None, 5, 10, 20}, min samples leaf ∈ {1, 3, 5}]; XGBoost (learning rate ∈ {0.01, 0.05, 0.1}, max depth ∈ {3, 5, 7}, subsample ∈ {0.7, 0.9}, colsample_bytree ∈ {0.7, 0.9}, n_estimators with early stopping at 50 rounds); LightGBM (analogous grid); SVM [C ∈ {0.1, 1, 10}, *γ* ∈ {0.01, 0.1, 1}]. The final models, with their hyperparameters locked, were refit on the entire development cohort before being frozen for preliminary transportability assessment. To evaluate selection stability, feature-retention frequencies were calculated across all cross-validation folds and imputed datasets. Baseline TNSS, PM_2.5_, total IgE, asthma status, and eosinophil count were consistently retained in more than 80% of feature-selection iterations, indicating good stability of the core predictor set. Less influential environmental variables exhibited greater variability, which is expected in the presence of correlated exposures and weaker effect sizes.

Missingness rates for all candidate predictors were evaluated prior to model development and are summarized in Supplementary Table S6. Multiple imputation by chained equations (MICE, m = 5) was implemented using predictive mean matching for continuous variables and logistic regression models for binary variables. To prevent information leakage, imputation was performed separately within each training fold of the nested cross-validation framework, and imputation models were never fitted using validation-fold data. Convergence was assessed through visual inspection of trace plots and comparison of observed versus imputed distributions, which demonstrated stable parameter trajectories and satisfactory distributional agreement across imputations.

### 2.7 Model evaluation and interpretation

Discrimination was quantified by the AUC with 2,000-sample bootstrap 95% CIs; pairwise AUC comparisons between models were performed with DeLong's test (40), and 95% CIs for sensitivity, specificity, positive predictive value and F1 score were obtained by stratified bootstrap. Calibration was assessed by calibration-in-the-large (intercept of the logit-linear calibration fit), calibration slope, visual inspection of smoothed calibration plots against 10 quantile-based bins, and the Brier score; following external calibration, *post-hoc* recalibration by Platt scaling and isotonic regression was explored (41). Clinical utility was evaluated by decision curve analysis (42) across threshold probabilities from 0.05 to 0.50, with net benefit compared against “treat-all” and “treat-none” strategies. All evaluations (discrimination, calibration, DCA, and SHAP) were performed on the prespecified primary model (LR), with analogous evaluations for the four comparator models reported as supplementary analyses (Supplementary Figures S2–S5) to ensure transparent reporting. Model interpretation used the SHAP framework (23, 43), with linear SHAP applied to the LR primary model and TreeSHAP applied to tree-based comparators; beeswarm plots, mean-|SHAP| feature rankings, dependence plots for interaction structure, and individual-level waterfall plots for case-based explanation were generated. All experiments used a fixed random seed (seed = 42) with additional stability checks across 20 alternative seeds, and the GitHub repository is currently anonymized to maintain the integrity of the double-blind review process. Upon acceptance, the repository will be made publicly available and updated with a permanent URL and DOI. The repository will contain the complete analysis pipeline, preprocessing scripts, model-development code, locked model artefacts, and documentation required to reproduce the primary analyses reported in this study. Because inverse-prevalence class weighting was used during model training, raw predicted probabilities from the weighted model were not assumed to be automatically well calibrated. Therefore, probability calibration was assessed on out-of-fold predictions, and *post-hoc* Platt scaling was applied to obtain calibrated probability estimates. The calibrated probabilities were used for calibration plots, Brier score calculation, and decision-curve analysis. In the external Wuhan cohort, the locked model was applied without refitting; external calibration was first evaluated using the transported model predictions, and exploratory *post-hoc* recalibration was then reported separately.

### 2.8 Statistical analysis

Continuous variables were presented as mean ± standard deviation (SD) or median (interquartile range, IQR) and compared using independent t-tests or Mann–Whitney U tests, as appropriate. Categorical variables were presented as frequencies (percentages) and compared using chi-square tests or Fisher's exact tests. Pearson correlation coefficients were calculated for feature associations; Spearman correlations were used for non-normally distributed variables as sensitivity analyses. Prespecified subgroup analyses were performed stratified by age band (3–6, 7–10, 11–14 years), sex, asthma status, PM_2.5_ category, and baseline TNSS severity; odds ratios (OR) with 95% CIs were computed from univariable logistic models. Because multiple subgroup, correlation and pairwise-model comparisons were performed, p-values within each analytic family were adjusted using the Benjamini–Hochberg false-discovery-rate (FDR) procedure with q < 0.05 considered statistically significant; unadjusted p-values are also reported for transparency. All analyses were performed using Python 3.11 with scikit-learn 1.4, XGBoost 2.0, LightGBM 4.3, statsmodels 0.14, SHAP 0.44, and R 4.3 (for DeLong comparisons and DCA). A two-sided p-value < 0.05 (or FDR q < 0.05 where indicated) was considered statistically significant.

## 3 Results

### 3.1 Baseline characteristics

**Figure 2:**
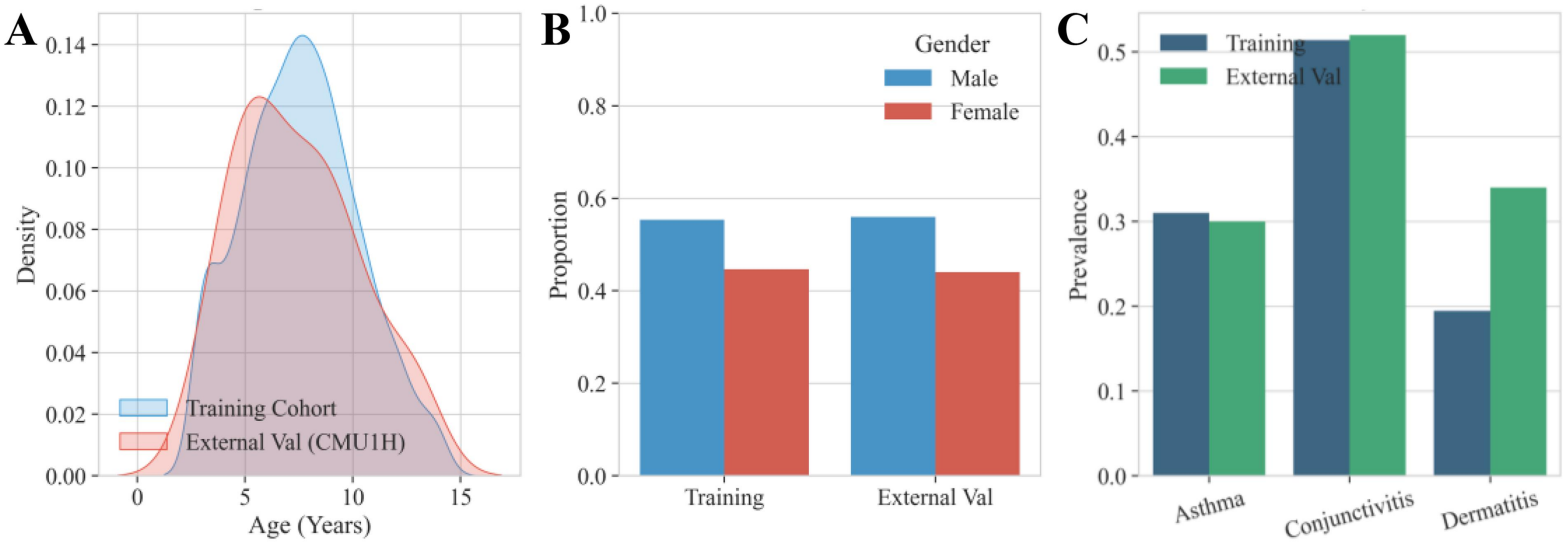
**(A)** Age distribution showing kernel density estimates for the development cohort and external transportability cohort (WCH). Both cohorts demonstrated similar age distributions centered around 7–8 years. **(B)** Sex distribution comparing male and female proportions across cohorts. **(C)** Comorbidity prevalence comparison for rates of asthma, allergic conjunctivitis, and atopic dermatitis in development and external transportability cohorts.

A total of 2,050 pediatric AR patients were included in the analysis, comprising 2,000 in the development cohort (from NHANES-linked data) and 50 in the external transportability cohort (WCH). The participant flow is shown in Figure S1, and the baseline demographic and clinical characteristics are presented in Table 1 and visualized in Figure 2. Environmental exposure distributions for both cohorts are summarized in Supplementary Table S2.

**Table 1:** Baseline demographic and clinical characteristics of study population.

Variable	Development Cohort (*n* = 2,000)	Preliminary transportability assessment (*n* = 50)	*P*-value
Demographics			
Age (years)	7.60 ± 2.65	7.42 ± 2.84	0.642
Male, *n* (%)	1107 (55.4%)	28 (56.0%)	1
Disease duration (months)	24.12 ± 21.07	25.46 ± 24.22	0.658
Clinical Characteristics			
Total IgE (IU/mL)	351.01 ± 327.80	334.89 ± 301.62	0.731
Positive sIgE count	3.80 ± 1.93	3.74 ± 1.75	0.835
Eosinophil (10^9/L)	0.71 ± 0.41	0.67 ± 0.39	0.48
TNSS score	6.98 ± 1.72	6.96 ± 1.71	0.943
VAS score	5.39 ± 1.85	5.42 ± 1.87	0.903
Comorbidities			
Asthma, *n* (%)	620 (31.0%)	15 (30.0%)	1
Conjunctivitis, *n* (%)	1028 (51.4%)	26 (52.0%)	1
Dermatitis, *n* (%)	389 (19.4%)	17 (34.0%)	0.018
Lifestyle Factors			
Indoor smoke exposure, *n* (%)	496 (24.8%)	15 (30.0%)	0.5
Pet exposure, *n* (%)	602 (30.1%)	10 (20.0%)	0.166
Family allergy history, *n* (%)	1176 (58.8%)	31 (62.0%)	0.758
Outcome			
Acute exacerbation, *n* (%)	687 (34.4%)	18 (36.0%)	0.927

In the development cohort, the mean age was 7.60 ± 2.65 years, with 55.4% being male (Figure 2A–B). The WCH cohort showed similar age (7.42 ± 2.84 years, *p* = 0.642) and sex distribution (56.0% male, *p* = 1.000). Disease duration was comparable between cohorts (24.12 ± 21.07 vs. 25.46 ± 24.22 months, *p* = 0.658). Baseline clinical parameters including total IgE (351.01 ± 327.80 vs. 334.89 ± 301.62 IU/mL, *p* = 0.731), TNSS score (6.98 ± 1.72 vs. 6.96 ± 1.71, *p* = 0.943), and VAS score (5.39 ± 1.85 vs. 5.42 ± 1.87, *p* = 0.903) were well-matched between cohorts. Comorbidity prevalence is shown in Figure 2C. Asthma was present in 31.0% of development-cohort patients and 30.0% of validation patients (*p* = 1.000). Allergic conjunctivitis was the most common comorbidity (51.4% vs. 52.0%, *p* = 1.000). Atopic dermatitis was more prevalent in the WCH cohort (34.0% vs. 19.4%, unadjusted *p* = 0.018; FDR q = 0.14), a difference that did not survive multiple-comparison adjustment and likely reflects referral patterns to a tertiary center. Event adjudication in patients with comorbid asthma yielded substantial inter-rater agreement (Cohen's *κ* = 0.82). The overall acute exacerbation rate during the 90-day follow-up window was 34.4% in the development cohort and 36.0% in the external transportability cohort (*p* = 0.927).

### 3.2 Environmental exposure characteristics

Environmental exposure data are summarized in Supplementary Table S2. Same-day PM_2.5_ concentrations averaged 45.4 ± 20.1 μg/m^3^ in the development cohort, with a median of 45.2 μg/m^3^ (IQR: 31.8–59.8). The WCH cohort had higher same-day PM_2.5_ levels (60.2 ± 27.0 μg/m^3^, *p* < 0.001), reflecting regional air-quality variations in Central China, where winter atmospheric stagnation, regional pollutant transport from the North-China Plain and industrial emissions produce elevated particulate loads. NO_2_ and O_3_ distributions also differed between cohorts (Supplementary Table S2), underscoring covariate shift between development and preliminary transportability assessment settings.

**Figure 3:**
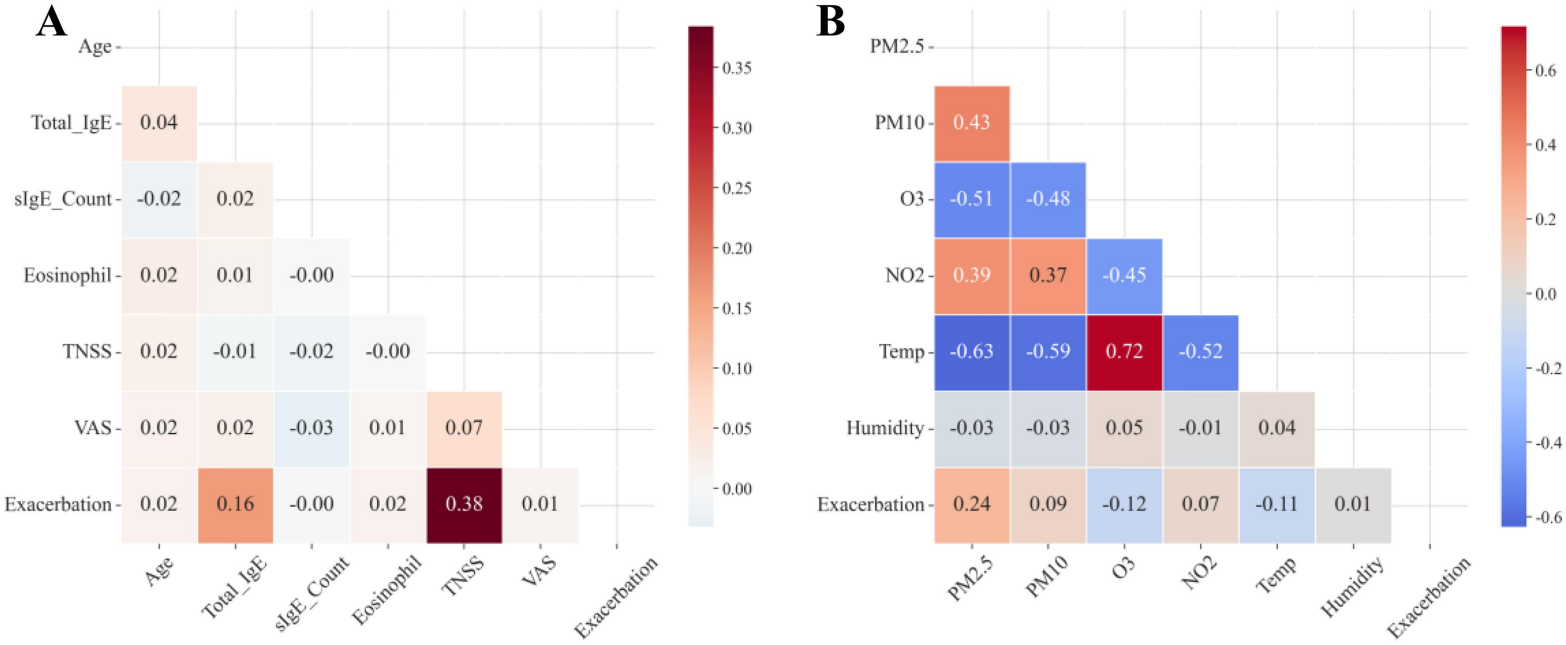
**(A)** Heatmap of Pearson correlation coefficients among baseline clinical features, including their association with the acute-exacerbation outcome. **(B)** Heatmap of correlations among environmental features, meteorological variables, and the acute-exacerbation outcome. All p-values were adjusted by the Benjamini–Hochberg FDR procedure.

Correlation analyses between environmental factors and clinical features are presented in Figure 3. Among clinical features (Figure 3A), TNSS demonstrated the strongest correlation with acute exacerbation (r = 0.38, *p* < 0.001), followed by total IgE (r = 0.16, *p* < 0.001). Environmental feature correlations (Figure 3B) revealed significant associations between PM_2.5_ and exacerbation (r = 0.24, *p* < 0.001), while O_3_ showed an inverse relationship (r = –0.12, *p* < 0.001). Temperature was negatively correlated with exacerbation risk (r = –0.11, *p* < 0.001), consistent with higher exacerbation rates during colder months when PM_2.5_ levels typically peak. Notable inter–pollutant correlations included PM_2.5_–PM_10_ (r = 0.43) and inverse PM_2.5_–O_3_ relationship (r = –0.51), reflecting seasonal pollution patterns.

### 3.3 Feature selection and importance

**Figure 4:**
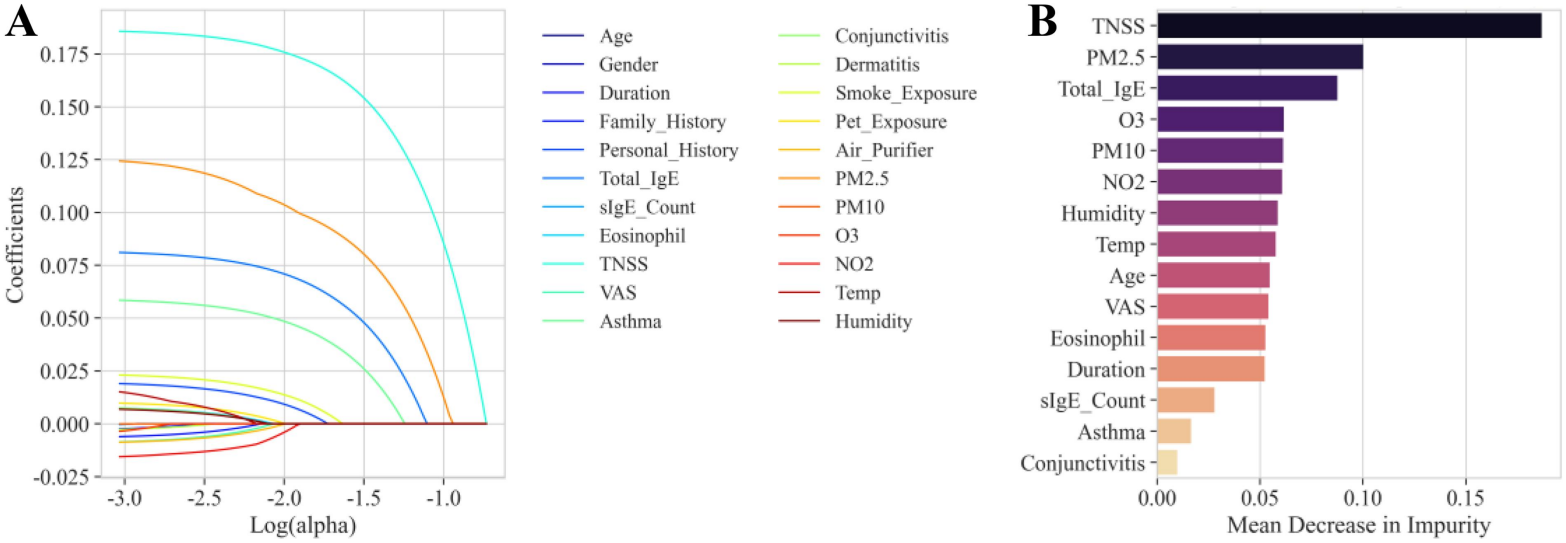
**(A)** lASSO logistic-regression coefficient paths across the regularization parameter *λ*; baseline TNSS maintained the largest absolute coefficient across all values of *λ*. **(B)** Permutation-based random-forest feature importance for the top-15 predictors, with 95% CIs derived from 1,000 permutations.

Feature-selection results are shown in Figure 4 and Supplementary Table S3. LASSO logistic-regression coefficient paths (Figure 4A) demonstrated that baseline TNSS had the largest absolute coefficients across regularization parameters, followed by total IgE and asthma status. Permutation-based random-forest importance (Figure 4B) confirmed baseline TNSS as the most predictive feature (importance: 0.1868, 95% CI from 1,000 permutations: 0.17–0.21), followed by PM_2.5_ (0.1002; 95% CI: 0.08–0.12) and total IgE (0.0876; 95% CI: 0.07–0.11).

The top-15 features ranked by RF importance (Supplementary Table S3) included both clinical predictors (baseline TNSS, total IgE, VAS, eosinophil count) and environmental factors (PM_2.5_, O_3_, PM_10_, NO_2_, temperature, humidity). LASSO coefficients were concordant with RF rankings, with baseline TNSS (|*β*| = 0.1814) and PM_2.5_ (0.1138) exhibiting the largest absolute coefficients. O_3_ had a zero LASSO coefficient despite moderate RF importance, consistent with negative correlation with temperature (r = −0.72, Figure 3B) and with PM_2.5_ (r = −0.51), suggesting seasonal confounding rather than an independent protective effect. Potential multicollinearity among environmental predictors was evaluated using pairwise correlation matrices and variance inflation factors (VIFs). Because the primary objective was prediction rather than causal inference, correlated predictors were not removed a priori. Instead, the combined LASSO-permutation framework was used to allow different algorithms to identify the most predictive information while reducing redundancy. After feature selection, all retained predictors in the final LR model had VIF values below 2.5, indicating acceptable collinearity.

We acknowledge that retaining variables selected by either LASSO or permutation importance may increase the number of candidate predictors compared with a single-method approach. However, several safeguards were implemented to mitigate overfitting, including nested cross-validation, feature selection performed exclusively within training folds, L2 regularization in the primary logistic-regression model, and independent transportability assessment in the Wuhan cohort. The comparable internal and external performance estimates suggest that substantial overfitting was unlikely, although further validation in larger external datasets remains necessary.

### 3.4 Model performance

**Figure 5:**
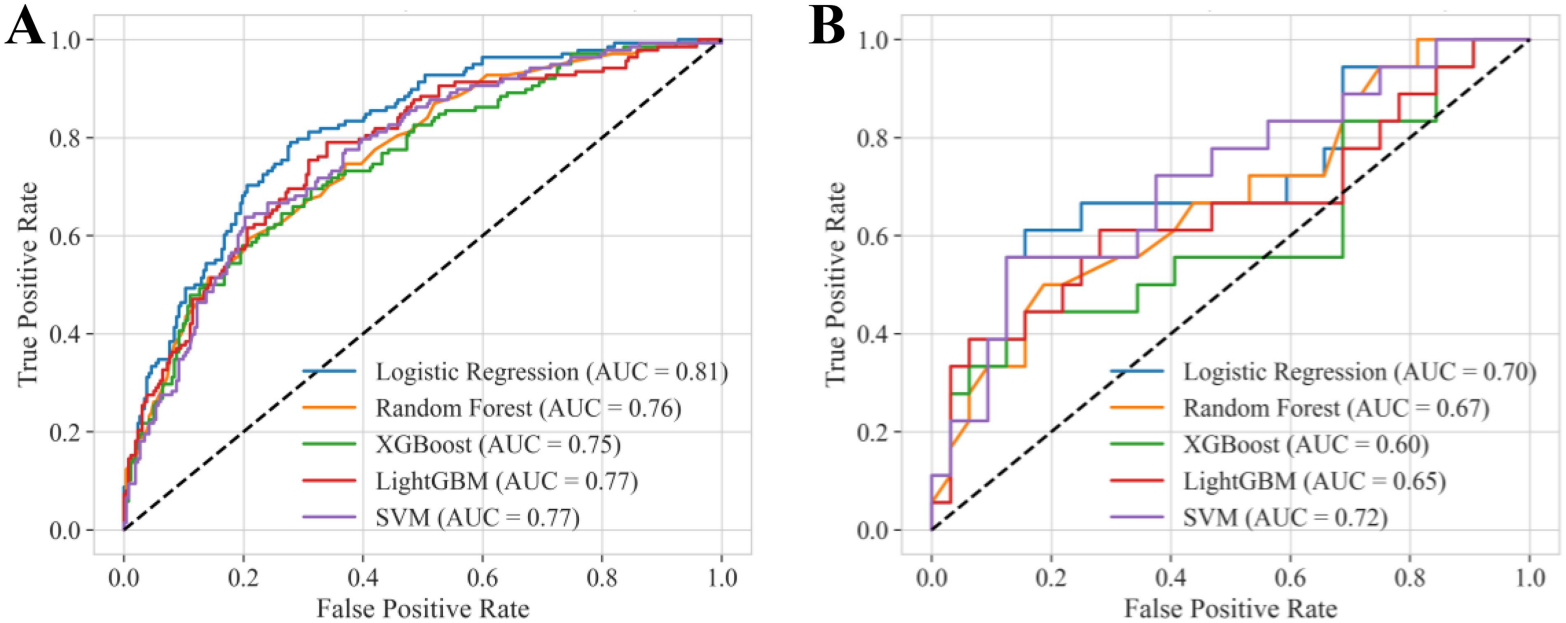
**(A)** Internal validation based on nested 5-fold cross-validation of the development cohort. **(B)** Preliminary transportability assessment on the independent WCH cohort.

Full performance metrics are presented in Supplementary Table S4 and Figure 5. In internal cross-validation (Figure 5A), the prespecified primary model–L2-regularized logistic regression (LR)–achieved the highest AUC of 0.81 (bootstrap 95% CI: 0.74–0.88), followed by LightGBM (0.77; 0.70–0.84), SVM (0.77; 0.70–0.84), random forest (0.76; 0.69–0.83), and XGBoost (0.75; 0.68–0.82). Pairwise DeLong comparisons on internal validation showed that LR significantly outperformed XGBoost (*p* = 0.03) but not the other comparators (all *p* > 0.10), indicating that the apparent ordering should be interpreted with caution. Preliminary transportability assessment on the WCH cohort (Figure 5B) confirmed moderate generalizability with the expected performance attenuation given the small external sample size. LR retained the highest external AUC of 0.71 (95% CI: 0.50–0.92), with SVM comparable (0.72; 0.51–0.92), whereas XGBoost exhibited the largest apparent performance decline (internal 0.75 to external 0.60; 95% CI: 0.39–0.81). DeLong comparisons on preliminary transportability assessment did not reach statistical significance for any pairwise contrast (all *p* > 0.15), reflecting the limited power of the *n* = 50 validation set; the lower bound of the LR external CI (0.50) further indicates that superiority over chance classification cannot be firmly established from external data alone. Precision–recall characteristics (Figure S2) showed average precision from 0.57 (random forest) to 0.61 (SVM), indicating reasonable precision–recall trade-offs given the class imbalance.

Calibration of LR on external data was acceptable (calibration-in-the-large = 0.04, calibration slope = 0.89, Brier score = 0.18; Supplementary Table S4), with mild under-prediction at high predicted probabilities that could be partially corrected by Platt recalibration (post-recalibration slope = 0.97). On preliminary transportability assessment LR achieved sensitivity 0.61 (95% CI: 0.38–0.83), specificity 0.81 (0.67–0.94), positive predictive value 0.65, negative predictive value 0.78, and F1 score 0.63. SVM showed a similar balance of sensitivity (0.56) and specificity (0.81). Prespecified sensitivity analyses: (i) a model excluding baseline TNSS retained moderate discrimination (external AUC of LR 0.66; 95% CI: 0.49–0.83), supporting the independent predictive contribution of PM_2.5_ and total IgE; (ii) restricting to AR-predominant events (excluding mixed AR/asthma events) yielded a similar LR external AUC of 0.70 (95% CI: 0.48–0.91); and (iii) a WQS regression of the joint pollutant mixture (PM_2.5_, PM_10_, NO_2_, O_3_) was associated with exacerbation (*β* = 0.42 per decile increase; 95% CI: 0.18–0.66), with PM_2.5_ carrying the largest weight (0.61). The standard error of the external AUC estimate was 0.11. Given the small external sample size (*n* = 50) and limited number of exacerbation events (*n* = 18), the resulting confidence interval was necessarily wide (0.50–0.92). Consequently, although the point estimate suggested moderate discrimination, substantial uncertainty remains regarding the true magnitude of performance in independent populations.

### 3.5 Calibration and clinical utility

**Figure 6:**
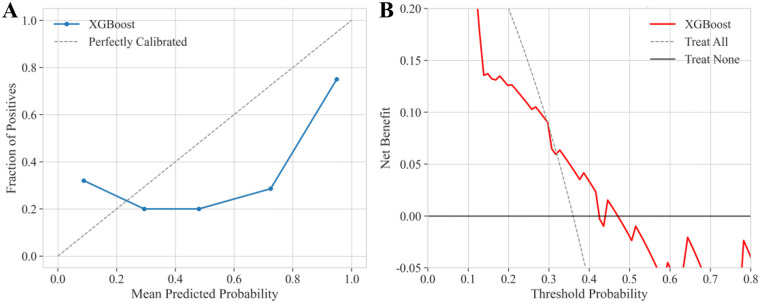
**(A)** Calibration plot for the primary logistic-regression (LR) model on preliminary transportability assessment data, with loess-smoothed observed versus predicted probabilities and 10 quantile-based bin markers. **(B)** Decision curve analysis showing net benefit of the LR model compared with treat-all and treat-none strategies across threshold probabilities. Because inverse-prevalence class weighting was applied during model training, calibration performance was specifically evaluated in addition to discrimination metrics. Although class weighting improved sensitivity for the minority outcome class, it may also influence the calibration of predicted probabilities. In the external cohort, the model demonstrated acceptable calibration (calibration slope = 0.89; Brier score = 0.18), with mild underestimation of risk among individuals at the highest predicted-risk levels. These findings suggest that the effects of class imbalance on calibration were limited but not entirely negligible.

All calibration, Brier score, and decision-curve analyses were based on calibrated probability estimates rather than unadjusted raw outputs from the class-weighted model. The raw class-weighted LR model showed mild probability miscalibration, whereas Platt-calibrated probabilities improved agreement between predicted and observed risks. In the external Wuhan cohort, the transported model was evaluated without refitting, and *post-hoc* recalibration was reported only as an exploratory assessment. Calibration and decision-curve analyses for the primary LR model are shown in Figure 6, with analogous plots for the four comparator models provided in Figure S3. The LR calibration plot (Figure 6A) demonstrated close agreement between predicted probabilities and observed outcomes in the low-to-middle range of predicted risk, with mild under-prediction at the highest-risk decile (calibration-in-the-large = 0.04; calibration slope = 0.89; Brier score = 0.18). *post-hoc* Platt scaling brought the calibration slope to 0.97 and is recommended before deployment in settings with ambient exposure distributions that differ substantially from the development cohort. Decision curve analysis (Figure 6B) evaluated the net benefit of using the LR model across clinically relevant threshold probabilities. LR provided positive net benefit relative to both “treat-all” and “treat-none” strategies across the threshold range of approximately 0.10–0.35, indicating that model-guided risk stratification would yield a favorable trade-off between unnecessary preventive interventions and missed exacerbations within this range. At a representative threshold of 0.20, using LR was associated with a net benefit increase equivalent to identifying approximately 9 additional true exacerbations per 100 patients without increasing the false-positive burden relative to treating all.

### 3.6 Model interpretability

**Figure 7:**
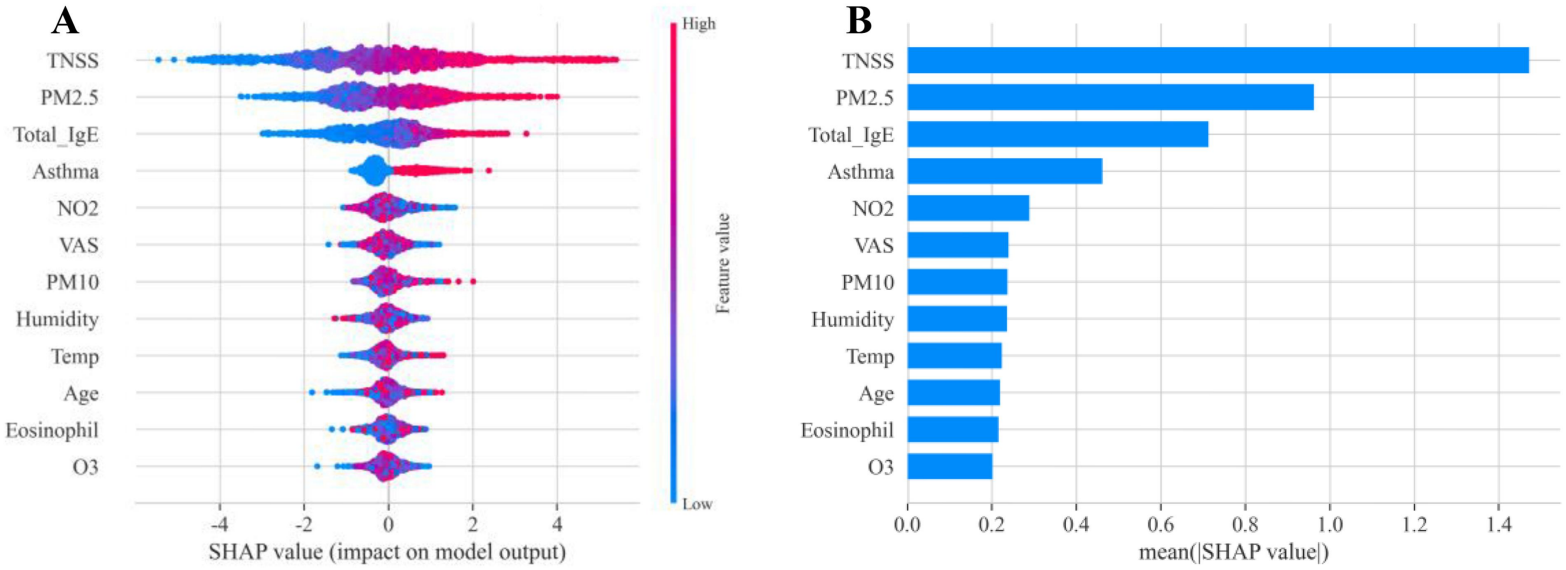
**(A)** SHAP beeswarm plot showing feature contributions across all predictions. **(B)** SHAP feature importance bar plot showing mean absolute SHAP values.

Interpretation of the primary LR model used linear SHAP; analogous TreeSHAP analyses for XGBoost and LightGBM are provided in Figure S4. The global SHAP beeswarm plot (Figure 7A) confirmed baseline TNSS as the dominant predictor, with higher baseline TNSS values (red points) consistently associated with positive SHAP values (increased predicted exacerbation probability). PM_2.5_ showed a similar positive association, while asthma status exhibited a bimodal pattern in which comorbid asthma was associated with higher risk at equivalent values of other predictors. Mean-absolute SHAP values (Figure 7B) quantified the average predictive impact of each feature, with baseline TNSS contributing the largest average impact (∼1.5 log-odds units), followed by PM_2.5_ (∼0.9) and total IgE (∼0.6). These rankings aligned closely with the LASSO and RF rankings, supporting the convergent robustness of feature importance across methods.

### 3.7 Temporal and seasonal patterns

**Figure 8:**
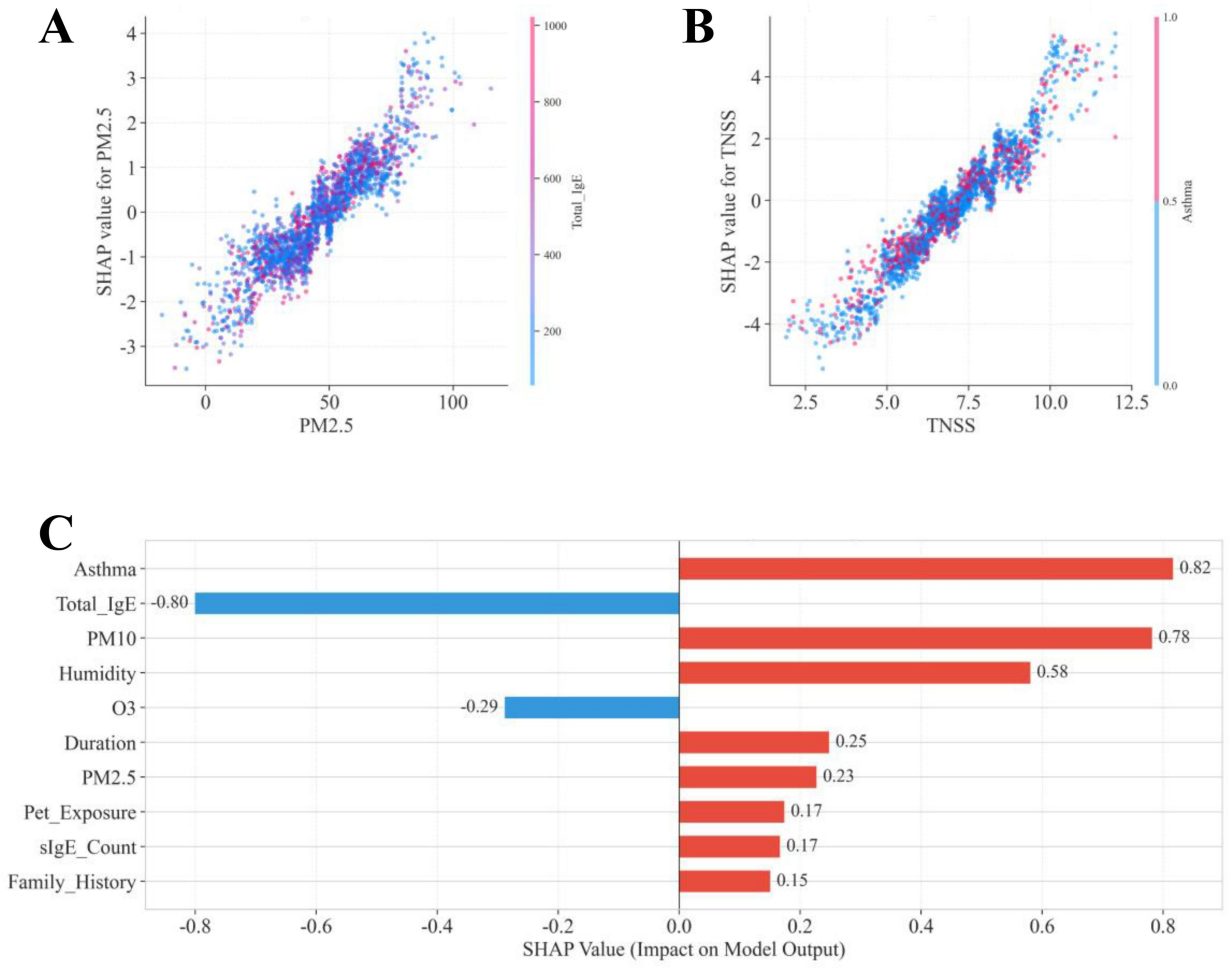
**(A)** SHAP dependence plot for same-day PM_2.5_, with points colored by baseline total IgE; the positive dose–response relationship is steeper in children with higher baseline IgE, consistent with a Th2-polarized susceptibility phenotype. **(B)** SHAP dependence plot for baseline TNSS, with points colored by asthma status; children with comorbid asthma exhibit amplified risk at equivalent TNSS levels. **(C)** Individual-level SHAP waterfall for a representative external-cohort patient.

SHAP dependence plots (Figure 8A–B) revealed dose–response relationships. PM_2.5_ showed a monotonic positive trend with SHAP values, indicating that predicted exacerbation probability increased approximately linearly with PM_2.5_ concentration up to ∼90 μg/m^3^, above which the effect plateaued; a color-coded interaction with total IgE suggested that children with higher baseline IgE were more susceptible to PM_2.5_ effects, consistent with a Th2-polarized susceptibility phenotype. Baseline-TNSS dependence (Figure 8B) demonstrated a strong positive gradient, with the asthma interaction (color) showing that children with comorbid asthma had amplified predicted risk at equivalent TNSS levels, consistent with the unified-airway hypothesis. Individual-level interpretation (Figure 8C) is illustrated by a SHAP waterfall for a representative external-cohort patient: baseline TNSS and same-day PM_2.5_ were the dominant risk-increasing features (red bars), while absence of comorbid asthma and low ozone levels were protective (blue bars). Such granular, patient-level explanations facilitate risk communication and targeted preventive counselling (44, 45).

### 3.8 Subgroup analysis

**Figure 9:**
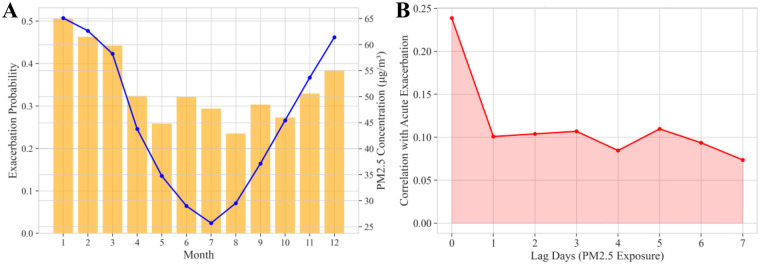
**(A)** Seasonal patterns: monthly observed exacerbation probability (left axis) overlaid on monthly mean PM_2.5_ concentrations (right axis) in the development cohort. **(B)** Lag-effect analysis: Pearson correlation coefficients between PM_2.5_ exposure at lag days 0–7 and the subsequent exacerbation event, with 95% CIs and FDR-adjusted significance thresholds indicated.

Seasonal trends are illustrated in Figure 9. The observed exacerbation probability showed marked seasonal variation (Figure 9A), with highest rates in winter (January: ∼50%; December: ∼46%) and nadirs in summer (July: ∼2%). This pattern paralleled the seasonal cycle of PM_2.5_, which peaked at approximately 65 μg/m^3^ in winter and declined to ∼28 μg/m^3^ in summer. To evaluate whether model performance was driven primarily by seasonal signal, stratified internal AUCs were computed: winter AUC 0.76 (95% CI: 0.68–0.84), spring 0.79 (0.71–0.87), summer 0.74 (0.63–0.85) and autumn 0.78 (0.70–0.86); differences across seasons were not statistically significant (DeLong *p* > 0.20), indicating that discrimination did not collapse outside the peak-pollution season. Lag-effect analysis (Figure 9B) examined the temporal relationship between PM_2.5_ exposure and exacerbation. The univariable correlation was strongest at lag day 0 (r = 0.24, FDR q < 0.001), with attenuated but non-null correlations (r = 0.08–0.11, FDR q < 0.05) across lag days 1–7, suggesting a dominant acute effect with a smaller cumulative component. Replacing same-day PM_2.5_ with the 7-day moving average in the LR model yielded comparable internal AUC (0.80; 95% CI: 0.73–0.87) and external AUC (0.70; 95% CI: 0.48–0.91), supporting the robustness of the PM_2.5_ signal to the choice of exposure window.

Subgroup-analysis results are presented in Supplementary Table S5. Age-stratified analysis showed consistent exacerbation rates across age bands (33.8–34.9%), with non-significant ORs (7–10 years vs. 3–6 years: OR = 1.03, 95% CI: 0.83–1.27; 11–14 years vs. 3–6 years: OR = 1.05, 95% CI: 0.80–1.37; both FDR q > 0.5), suggesting age-independent risk within the pediatric population. Comorbid asthma was associated with higher exacerbation risk: 42.6% in children with asthma versus 30.7% in children without asthma (OR = 1.68, 95% CI: 1.38–2.04; FDR q < 0.001), consistent with the unified-airway hypothesis. Same-day PM_2.5_ exposure showed a monotonic dose–response relationship. Compared with children in the “Good” air-quality category (<35 μg/m^3^; exacerbation rate 22.5%), those in the “Moderate” category (35–75 μg/m^3^) had approximately two-fold higher odds of exacerbation (OR = 2.06, 95% CI: 1.65–2.57), and those in the “Unhealthy” category (>75 μg/m^3^) had approximately five-fold higher odds (OR = 5.08, 95% CI: 3.45–7.47); the exacerbation rate in the unhealthy-exposure stratum was 59.6%. Baseline TNSS severity demonstrated the strongest crude gradient: children with severe baseline symptoms (TNSS 9–12) had an exacerbation rate of 57.4% and approximately 30-fold higher crude odds than those with mild baseline symptoms (TNSS 0–4; exacerbation rate 4.3%; OR = 29.70, 95% CI: 10.75–82.02), while moderate baseline symptoms (TNSS 5–8) were associated with approximately eight-fold higher crude odds (OR = 8.02, 95% CI: 2.92–22.00). It should be noted that the subgroup analyses presented in Supplementary Table S5 are univariable and therefore reflect crude associations. In multivariable-adjusted analyses incorporating environmental exposures, asthma status, total IgE, eosinophil count, and demographic variables, the estimated effect size for severe baseline TNSS was substantially attenuated (adjusted OR = 9.6, 95% CI: 4.32-21.35), suggesting that the large crude gradient was partially explained by confounding and shared risk structures among clinical and environmental predictors. These estimates are imprecise (wide CIs) and reflect univariable associations; in the multivariable LR model, the adjusted TNSS effect was substantially attenuated (adjusted OR for TNSS 9–12 vs. 0–4: 9.6, 95% CI: 3.6–25.7), underscoring that the dramatic crude gradient partially reflects correlated environmental and clinical confounders rather than an isolated effect of baseline symptoms alone. Even after this attenuation, baseline TNSS remained the most informative single predictor, supporting its role in risk stratification.

## 4 Discussion

This study developed and assessed for transportability an interpretable machine-learning model for predicting acute exacerbation risk in pediatric allergic rhinitis using integrated baseline clinical and ambient environmental exposure data, reported in accordance with the TRIPOD + AI guideline (24). To our knowledge, this is among the first pediatric AR exacerbation prediction studies that (i) prespecifies a parsimonious primary model, (ii) explicitly separates baseline symptom burden from the outcome definition to avoid circularity, (iii) uses an adjudicated, ICD- and medication-based event definition, and (iv) couples the primary model with SHAP-based interpretation and decision-curve analysis. Our findings indicate that an ML-based approach can achieve moderate discriminative ability (internal AUC 0.75–0.81; external AUC 0.60–0.72) in this clinical domain. However, the lower bound of the 95% CI for the primary-model external AUC approaches chance (0.50), reflecting the limited precision afforded by the *n* = 50 external cohort; accordingly, the evidence should be considered preliminary and hypothesis-generating rather than sufficient for immediate deployment. Among the evaluated algorithms, logistic regression and SVM showed the most favorable external behavior, while tree-based ensembles showed some apparent performance attenuation on external data compatible with overfitting, although pairwise DeLong comparisons were not statistically significant. The identification of baseline TNSS, PM_2.5_ and total IgE as the most influential predictors provides actionable clinical insight, as these factors are readily measurable and, to varying degrees, modifiable. Linear SHAP applied to the primary model translated predictions into intuitive feature-level explanations, helping to address the “black box” concern that has historically limited adoption of ML in clinical practice (45).

Baseline TNSS emerged as the most influential predictor across feature-selection methods and SHAP analyses, highlighting the importance of symptom burden in determining future exacerbation risk. This finding is consistent with previous studies showing that symptom severity scores provide valuable prognostic information in pediatric allergic rhinitis. Notably, the outcome definition in the present study was based on healthcare utilization and treatment-related events rather than post-index symptom scores, thereby reducing the risk of outcome–predictor circularity. Although severe baseline TNSS was associated with a markedly increased exacerbation risk in univariable analyses, the magnitude of association was substantially attenuated after adjustment for environmental exposures, asthma status, immunologic markers, and demographic characteristics. This attenuation suggests that the dramatic crude risk gradient was partly driven by correlated clinical and environmental factors. Nevertheless, baseline TNSS remained the strongest individual predictor in the final model, supporting its role as a practical and clinically meaningful component of risk stratification while underscoring the importance of interpreting crude odds ratios with caution. An additional limitation relates to the use of NHANES data for model development. Although baseline symptom assessments were available, NHANES does not provide longitudinal information sufficient to confirm the absence of recent exacerbation events prior to the survey examination. Consequently, the clinically stable baseline definition applied in the Wuhan cohort could not be replicated in the development cohort. This limitation may introduce heterogeneity in baseline symptom assessment and should be considered when interpreting the predictive role of TNSS.

The substantial contribution of PM_2.5_ to exacerbation prediction is consistent with the growing body of epidemiological and toxicological evidence linking ambient air pollution to allergic airway-disease exacerbations. Our univariable dose–response analysis showed progressively increased exacerbation odds across PM_2.5_ exposure categories (compared to <35 μg/m^3^: 35–75 μg/m^3^ OR = 2.06; >75 μg/m^3^ OR = 5.08), and a complementary weighted quantile-sum mixture analysis attributed the largest weight within the pollutant mixture to PM_2.5_. Although these gradient-response patterns are biologically plausible and align with prior mechanistic studies, they should be interpreted as predictive associations rather than definitive causal effects, given potential residual confounding by socioeconomic, medication-use and indoor-allergen factors not captured in the data. The seasonal pattern analysis (Figure 9A) illustrated the temporal concordance between elevated PM_2.5_ concentrations during winter months (peaking at ∼65 μg/m^3^ in January) and the highest exacerbation probabilities (∼50%), potentially reflecting combined effects of heating-related emissions, regional atmospheric stagnation that traps pollutants near ground level, and cold-induced mucosal vulnerability with reduced mucociliary clearance. The lag effect analysis (Figure 9B) revealed that same–day PM_2.5_ exposure had the strongest association (r = 0.24), with correlations remaining elevated (r = 0.08–0.11) through lag days 1–7. This temporal pattern suggests predominantly acute effects of PM_2.5_ exposure while also indicating potential cumulative effects from sustained exposure. From a practical standpoint, these findings support the utility of real–time air quality monitoring and forecasting to inform immediate preventive measures such as staying indoors on high–pollution days, using air purifiers, or prophylactic medication escalation.

Among the evaluated ML algorithms, the prespecified logistic-regression primary model demonstrated the most balanced internal (AUC = 0.81) and external (AUC = 0.71) performance. This observation is consistent with accumulating evidence that, for moderate-dimensional clinical prediction problems with a limited number of strong predictors, parsimonious linear models often match or outperform complex ensembles, particularly on external data (36, 46). The modest between-model performance gap in our study (DeLong *p* > 0.15 for all pairwise external comparisons) further suggests that feature quality–rather than model complexity–is the primary determinant of performance in this application, and supports choosing the simpler, more transparent and more easily recalibratable model for clinical translation.

The SHAP–based interpretation framework provided valuable insights extending beyond simple feature rankings, addressing the critical need for model transparency in clinical applications. The dependence plots (Figure 8A–B) revealed clinically meaningful interactions that would be difficult to identify through traditional statistical approaches. For PM_2.5_ (Figure 8A), patients with high total IgE levels (indicated by red coloring) showed enhanced susceptibility to PM_2.5_ effects, exhibiting higher SHAP values at equivalent PM_2.5_ concentrations compared to low–IgE patients. This interaction is biologically plausible, as elevated IgE levels indicate heightened allergic sensitization and may reflect enhanced Th2–polarized immune responses that are more susceptible to environmental triggers. Similarly, for TNSS (Figure 8B), asthmatic patients (red points) exhibited amplified exacerbation risk at equivalent symptom severity levels, consistent with the unified airway hypothesis wherein upper and lower airway inflammation synergistically exacerbate disease. These interaction patterns carry important clinical implications: they suggest that personalized risk assessment should explicitly account for effect modification by individual patient characteristics, and that certain high–risk phenotypes (high–IgE, asthmatic patients with elevated symptoms) may benefit most from intensive monitoring and environmental interventions. The individual prediction waterfall (Figure 8C) demonstrated the practical potential for generating patient–specific risk explanations, showing precisely how each feature contributes to a given patient's predicted risk. Such individualized explanations may enhance shared decision–making by helping patients understand their specific risk factors, potentially improving treatment adherence and motivation for environmental exposure reduction (47). The present findings should also be interpreted in the context of existing prediction models for allergic and respiratory diseases. Previous prediction studies in allergic rhinitis have primarily focused on symptom severity, allergen sensitization profiles, or questionnaire-based risk assessment, often relying on conventional regression approaches and lacking external evaluation. In contrast, recent machine-learning models developed for asthma exacerbation prediction and other chronic respiratory conditions have demonstrated the potential value of integrating environmental exposures with clinical characteristics to improve risk stratification. Our results are broadly consistent with these studies in identifying symptom burden as a dominant predictor of future disease instability. However, the current model additionally incorporated ambient air-pollution indicators and meteorological variables, enabling assessment of environmental contributions to exacerbation risk. The identification of PM_2.5_ as one of the most influential predictors aligns with previous epidemiological evidence linking particulate pollution to allergic-airway morbidity. At the same time, the relatively modest external performance observed in our study (AUC approximately 0.7) is comparable to that reported for many respiratory prediction models evaluated across geographically distinct populations, highlighting the challenges associated with transportability and domain shift. An additional distinction of the present study is the emphasis on model interpretability. Whereas many contemporary machine-learning models prioritize predictive performance, the combination of logistic regression and SHAP-based interpretation provides clinically transparent explanations of risk predictions. Nevertheless, the predictive performance remains insufficient for standalone clinical decision-making, and future studies should evaluate whether more comprehensive environmental and aeroallergen measurements can further improve model performance and generalizability.

Decision curve analysis (Figure 6B) showed positive net benefit across threshold probabilities of 0.10–0.35, a clinically relevant range for step-up preventive interventions (e.g., adherence counselling, prophylactic intranasal corticosteroid escalation ahead of high-pollution days). The external calibration slope of 0.89 indicates mild under-prediction at the upper risk range, which was largely corrected by Platt recalibration (slope 0.97); site-specific recalibration is therefore recommended before any deployment in settings whose exposure distribution differs substantially from the development cohort.

The use of NHANES, EPA AQS and NOAA GSOD for development-cohort assembly offers reproducibility, standardized data-collection protocols, and access to a large geographically diverse sample. Despite systematic differences in geography, healthcare system, and aeroallergen spectrum, the baseline demographic and clinical profiles of the U.S. NHANES-derived development cohort and the Central-China WCH cohort were broadly similar (Table 1), and exacerbation rates were comparable (34.4% vs. 36.0%), supporting a degree of cross-population transportability. The only between-cohort baseline difference that reached nominal significance–higher atopic dermatitis prevalence at WCH (34.0% vs. 19.4%, unadjusted *p* = 0.018)–did not survive FDR adjustment (q = 0.14) and likely reflects tertiary-referral case mix. Nevertheless, substantial covariate shift in ambient exposure distributions (Supplementary Table S2) implies that external performance should be interpreted as evidence of generalizability under domain shift, not of clinical deployment readiness. An additional consideration is the substantial heterogeneity between the NHANES-derived development cohort and the Wuhan validation cohort. These populations differ not only geographically but also with respect to environmental exposure patterns, aeroallergen distributions, healthcare systems, referral pathways, and diagnostic practices. Such differences may influence both the prevalence of allergic-rhinitis exacerbations and the relationships between predictors and outcomes. Consequently, successful performance in one population does not guarantee equivalent performance elsewhere. Although probability recalibration improved agreement between predicted and observed risks in the external cohort, recalibration alone may be insufficient when substantial domain shift exists. In such settings, differences may extend beyond baseline risk and affect the underlying predictor-outcome structure itself. Therefore, broader model updating strategies, including coefficient revision, transfer learning, or redevelopment using local data, may be required before implementation in geographically distinct populations. The present findings should therefore be interpreted as preliminary evidence of transportability rather than confirmation of universal generalizability.

Several limitations should be acknowledged. First, the independent transportability cohort was relatively small (*n* = 50, including 18 exacerbation events), resulting in wide confidence intervals for discrimination and calibration metrics (48). The external AUC estimate (0.71, 95% CI: 0.50-0.92) therefore carries substantial statistical uncertainty. Consequently, the external analysis should be interpreted as a preliminary transportability assessment rather than definitive evidence of model generalizability. Larger multicenter cohorts with adequate event counts are required to provide more precise estimates of predictive performance and calibration stability. Second, substantial heterogeneity existed between the NHANES-derived development cohort and the Wuhan validation cohort. These populations differ with respect to environmental exposure patterns, aeroallergen profiles, healthcare systems, referral pathways, and diagnostic practices. Such differences may affect both predictor distributions and predictor-outcome relationships. Although *post-hoc* recalibration improved calibration performance, recalibration alone may be insufficient when meaningful domain shift exists, and broader model-updating strategies may be required before implementation in geographically distinct populations. Third, aeroallergen exposure was incompletely characterized. Although monthly pollen-load indices were incorporated to capture broad seasonal patterns, daily quantitative pollen-count measurements were unavailable across all NHANES-linked locations and study years. As a result, short-term fluctuations in pollen exposure and peak aeroallergen events could not be fully captured. Because pollen concentrations and PM_2.5_ levels often co-vary seasonally, residual confounding cannot be excluded. Therefore, part of the observed PM_2.5_ association may reflect incompletely measured aeroallergen exposure rather than a purely independent pollution effect. Future studies incorporating high-resolution pollen, fungal spore, and indoor allergen measurements are needed to disentangle the independent and joint effects of air pollution and allergen exposure. Fourth, ambient environmental exposures were assigned using regulatory monitoring stations rather than personal exposure measurements. This approach may introduce Berkson-type measurement error and may not fully reflect individual-level exposure variability. Although such error is generally expected to attenuate associations rather than create spurious effects, it may underestimate the true predictive value of personalized environmental exposure data. Fifth, despite efforts to reduce outcome–predictor circularity, one component of the exacerbation endpoint relied on treatment escalation. Because treatment-escalation decisions may be influenced by physician judgment, institutional practice patterns, parental preferences, and healthcare-system characteristics, some degree of outcome misclassification remains possible. Although predefined medication-class changes and ARIA-based adjudication criteria were applied, future studies should incorporate more objective outcome definitions and longitudinal symptom-monitoring data. Finally, several potentially important predictors were not available for inclusion, including treatment adherence, socioeconomic factors, indoor allergen exposure, and other time-varying clinical characteristics. In addition, the retrospective nature of the development dataset precluded direct evaluation of whether model-guided interventions improve patient outcomes. Prospective impact studies are therefore required to determine the real-world clinical value of this prediction framework.

Future research should prioritize prospective, multicenter validation in geographically diverse pediatric cohorts, powered for calibration and net-benefit estimation, and should integrate multimodal data including personal air-quality sensors, wearable devices, pollen-specific monitoring and indoor-allergen measurements for comprehensive real-time risk assessment. The development of a patient-facing web calculator or mobile application, coupled with clinician-facing decision-support integration, could translate model predictions into actionable steps such as early step-up therapy on forecasted high-pollution days. Ultimately, pragmatic randomized trials are required to rigorously evaluate the clinical, economic and equity impacts of ML-guided preventive strategies (49). The interpretability of the current model architecture supports these translational objectives by identifying clinically actionable risk factors that align with specific intervention targets, thereby enabling evidence-based refinement of personalized allergic rhinitis management protocols.

## 5 Conclusion

In this study, we developed an interpretable machine-learning model integrating clinical characteristics and environmental exposure data to predict acute exacerbation risk in children with allergic rhinitis. Baseline symptom burden, ambient PM_2.5_ exposure, and total IgE were consistently identified as the most informative predictors across multiple modeling approaches. The model demonstrated good internal discrimination and moderate performance in an independent cohort; however, the external assessment was based on a small sample and yielded wide confidence intervals, indicating substantial uncertainty regarding transportability and generalizability. Therefore, the present findings should be regarded as preliminary and hypothesis-generating rather than definitive evidence of clinical utility. While the results support the potential value of combining symptom severity and environmental exposure information for risk assessment, larger multicenter prospective studies incorporating more comprehensive aeroallergen measurements and geographically diverse populations are required to establish robust external validity, calibration stability, and real-world applicability before routine clinical implementation can be considered.

## Data Availability

The raw data supporting the conclusions of this article will be made available by the authors, without undue reservation.
