# Supplementary material for: Leveraging machine learning models to forecast pediatric allergic rhinitis exacerbation risk based on environmental exposure data
Source: Front Pediatr. 2026 Jul 22;14:1865399. doi: 10.3389/fped.2026.1865399 (PMC13437778; doi:10.3389/fped.2026.1865399)
Supplement: Supplementary file 1 [file Supplementaryfile1.docx]

**Supplementary materials**

**Leveraging Machine Learning Models to Forecast Pediatric Allergic Rhinitis Exacerbation Risk Based on Environmental Exposure Data**

Zhicheng Li^1^, Shan Huang^1^ and Zhongfang Xia^1,*^

^1^ Department of Otolaryngology, Wuhan Children’s Hospital, Tongji Medical College, Huazhong University of Science and Technology, Wuhan, Hubei, 430016, China

^*^ Corresponding author: Zhongfang Xia (E–mail: xiazhongfang_hust@163.com)

**Table S1.** TRIPOD+AI reporting checklist.

| **Item** | **Topic** | **Reported in** |
| --- | --- | --- |
| 1 | Title identifies AI/ML and prediction | Title; Abstract |
| 2 | Structured abstract (objective, methods, results, conclusions, registration) | Abstract |
| 3 | Background and rationale | section 1 Introduction |
| 4 | Specific objectives | section 1 (last paragraph), Abstract |
| 5 | Study design (retrospective cohort; development + external validation) | section 2.1 |
| 6 | Source of data (NHANES + EPA AQS + NOAA GSOD; WCH) | section 2.1 |
| 7 | Participants (eligibility, setting, dates) | section 2.1, section 2.2 |
| 8 | Outcome definition and ascertainment | section 2.5 |
| 9 | Predictors (how, when, and by whom measured) | section 2.3, section 2.4 |
| 10 | Sample size justification | section 2.1, section 4 (limitations); Ref 50 |
| 11 | Missing data handling (MICE, m = 5) | section 2.6 |
| 12 | Statistical analysis methods | section 2.6, section 2.7, section 2.8 |
| 13 | Development of the model (hyperparameters, CV, class imbalance) | section 2.6 |
| 14 | Specification of the final model (primary = LR) | section 2.6 (last sentence) |
| 15 | Model performance measures (AUC, calibration, Brier, DCA) | section 2.7 |
| 16 | Model updating/recalibration | section 2.7 (Platt/isotonic) |
| 17 | Analytical software and versions | section 2.8 |
| 18 | Participant flow (CONSORT-like) | Fig. S1 |
| 19 | Baseline characteristics | Table 1 |
| 20 | Model development results | section 3.3, section 3.4, Table S3, Table S4 |
| 21 | Model specification (weights/coefficients) | GitHub repository |
| 22 | Model performance on development and external data | section 3.4, Table S4 |
| 23 | Model interpretation (SHAP) | section 3.6, Fig. 7, Fig. 8, Fig. S4 |
| 24 | Discussion of limitations, biases, generalizability | section 4 (Limitations) |
| 25 | Clinical utility / net benefit | section 3.5, Fig. 6B |
| 26 | Data sharing / code availability | Data availability |
| 27 | Funding and conflicts | Declarations |

**Fig. S1.** CONSORT-like participant flow diagram.

Table S2. Environmental exposure characteristics.

| **Variable** | **Training Mean ± SD** | **Training Median (IQR)** | **External Val Mean ± SD** | **Correlation with Exacerbation (r)** | **P-value** |
| --- | --- | --- | --- | --- | --- |
| PM_2.5_ (ug/m^3^) | 45.4 ± 20.1 | 45.2 (31.8-59.8) | 60.2 ± 27.0 | 0.239 | <0.001 |
| PM_10_ (ug/m^3^) | 70.1 ± 26.6 | 69.8 (52.0-88.9) | 85.7 ± 37.6 | 0.09 | <0.001 |
| O_3_ (ug/m^3^) | 60.0 ± 25.9 | 61.0 (39.9-79.5) | 86.4 ± 28.5 | -0.122 | <0.001 |
| NO_2_ (ug/m^3^) | 40.1 ± 12.4 | 40.0 (31.2-48.7) | 44.1 ± 14.3 | 0.068 | 0.002 |
| Temperature (C) | 15.1 ± 11.7 | 15.6 (5.1-25.0) | 11.6 ± 13.1 | -0.114 | <0.001 |
| Relative Humidity (%) | 60.2 ± 12.2 | 60.0 (51.8-68.6) | 58.0 ± 16.3 | 0.015 | 0.508 |

Table S3. Feature importance rankings from multiple methods.

| **Rank** | **Feature** | **RF Importance** | **LASSO \|Coefficient\|** | **Correlation with Outcome** |
| --- | --- | --- | --- | --- |
| 1 | TNSS | 0.1868 | 0.1814 | 0.391 |
| 2 | PM_2.5_ | 0.1002 | 0.1138 | 0.239 |
| 3 | Total_IgE | 0.0876 | 0.0765 | 0.162 |
| 4 | O_3_ | 0.0615 | 0 | -0.122 |
| 5 | PM_10_ | 0.0612 | 0 | 0.09 |
| 6 | NO_2_ | 0.0608 | 0.0114 | 0.068 |
| 7 | Humidity | 0.0585 | 0.003 | 0.015 |
| 8 | Temp | 0.0577 | 0.0039 | -0.114 |
| 9 | Age | 0.0547 | 0 | 0.013 |
| 10 | VAS | 0.054 | 0.0042 | 0.01 |
| 11 | Eosinophil | 0.0526 | 0.0035 | 0.023 |
| 12 | Duration | 0.0523 | 0 | 0.012 |
| 13 | sIgE_Count | 0.0278 | 0 | -0.003 |
| 14 | Asthma | 0.0164 | 0.0541 | 0.116 |
| 15 | Conjunctivitis | 0.0098 | 0.0031 | 0.006 |

**Fig. S2.** Precision-Recall curves for external validation. Average precision ranged from 0.57-0.61 across models.

Table S4. Machine learning model performance comparison.

| **Model** | **Internal Validation AUC (95% CI)** | **External Validation AUC (95% CI)** | **Sensitivity** | **Specificity** | **PPV** | **NPV** | **F1-Score** |
| --- | --- | --- | --- | --- | --- | --- | --- |
| Logistic Regression | 0.81 (0.74-0.88) | 0.71 (0.50-0.92) | 0.61 | 0.81 | 0.65 | 0.79 | 0.63 |
| Random Forest | 0.76 (0.69-0.83) | 0.67 (0.46-0.89) | 0.39 | 0.84 | 0.58 | 0.71 | 0.47 |
| XGBoost | 0.75 (0.68-0.82) | 0.60 (0.37-0.83) | 0.44 | 0.72 | 0.47 | 0.7 | 0.46 |
| LightGBM | 0.77 (0.70-0.84) | 0.65 (0.43-0.87) | 0.56 | 0.72 | 0.53 | 0.74 | 0.54 |
| SVM | 0.77 (0.70-0.84) | 0.72 (0.51-0.92) | 0.56 | 0.81 | 0.62 | 0.76 | 0.59 |

**Fig. S3.** Calibration (top) and decision-curve analysis (bottom) for the four comparator models on the external validation cohort.

**Fig. S4.** TreeSHAP interpretability analyses for the XGBoost and LightGBM comparator models.

**Fig. S5.** Prespecified sensitivity analyses of the primary LR model on external validation.

Table S5. Subgroup analysis of acute exacerbation risk (univariable estimates).

| Subgroup | N | Exacerbation n (%) | OR (95% CI) |
| --- | --- | --- | --- |
| Age 3-6 years | 591 | 200 (33.8%) | - |
| Age 7-10 years | 1028 | 354 (34.4%) | 1.03 (0.83-1.27) |
| Age 11-14 years | 381 | 133 (34.9%) | 1.05 (0.80-1.37) |
| Without Asthma | 1380 | 423 (30.7%) | - |
| With Asthma | 620 | 264 (42.6%) | 1.68 (1.38-2.04) |
| PM_2.5_ Good (<35) | 618 | 139 (22.5%) | - |
| PM_2.5_ Moderate (35-75) | 1241 | 464 (37.4%) | 2.06 (1.65-2.57) |
| PM_2.5_ Unhealthy (>75) | 141 | 84 (59.6%) | 5.08 (3.45-7.47) |
| TNSS Mild (0-4) | 92 | 4 (4.3%) | - |
| TNSS Moderate (5-8) | 1344 | 359 (26.7%) | 8.02 (2.92-22.00) |
| TNSS Severe (9-12) | 564 | 324 (57.4%) | 29.70 (10.75-82.02) |

Table S6. Missingness summary and multivariable-adjusted associations of key predictors.

| **Predictor** | **Missing (%)** | **Adjusted OR** | **95% CI** | **P value** |
| --- | --- | --- | --- | --- |
| Severe TNSS (9-12 vs. 0-4) | 0 | 9.6 | 4.18-22.05 | <0.001 |
| Moderate TNSS (5-8 vs. 0-4) | 0 | 3.42 | 1.47-7.96 | 0.004 |
| PM_2.5_ Moderate (35-75 vs. <35 μg/m^3^) | 1.8 | 1.74 | 1.29-2.34 | <0.001 |
| PM_2.5_ Unhealthy (>75 vs. <35 μg/m^3^) | 1.8 | 3.21 | 1.87-5.52 | <0.001 |
| Total IgE (per SD increase) | 12.6 | 1.48 | 1.24-1.77 | <0.001 |
| Eosinophil count (per SD increase) | 8.9 | 1.26 | 1.08-1.47 | 0.003 |
| Asthma (Yes vs. No) | 0 | 1.71 | 1.29-2.27 | <0.001 |
| Conjunctivitis (Yes vs. No) | 0 | 1.18 | 0.91-1.53 | 0.211 |
| Age (per year) | 0 | 1.03 | 0.97-1.09 | 0.337 |
| NO_2_ (per SD increase) | 2.4 | 1.12 | 0.96-1.31 | 0.149 |
| O_3_ (per SD increase) | 2.4 | 0.89 | 0.76-1.05 | 0.172 |
| Temperature (per SD increase) | 1.7 | 0.91 | 0.79-1.06 | 0.221 |
| Relative humidity (per SD increase) | 1.5 | 1.04 | 0.90-1.20 | 0.613 |
